# Plant responses to butterfly oviposition partly explain preference–performance relationships on different brassicaceous species

**DOI:** 10.1007/s00442-019-04590-y

**Published:** 2020-01-13

**Authors:** Eddie Griese, Ana Pineda, Foteini G. Pashalidou, Eleonora Pizarro Iradi, Monika Hilker, Marcel Dicke, Nina E. Fatouros

**Affiliations:** 1 Laboratory of Entomology, Wageningen University, Wageningen, The Netherlands; 2 Present Address: Biosystematics Group, Wageningen University, Wageningen, The Netherlands; 3 Present Address: NIOO-KNAW, Wageningen, The Netherlands; 4 Present Address: UMR Agronomie, INRA, AgroParisTech, Universite Paris-Saclay, 78850 Thiverval-Grignon, France; 5 Present Address: BASF Chile, Carrascal 3851, Quinta Normal, Santiago, Chile; 6 Institute of Biology, Freie Universität Berlin, Berlin, Germany

**Keywords:** Hypersensitive response, Oviposition-induced, Egg-killing, Priming, Pieridae

## Abstract

The preference–performance hypothesis (PPH) states that herbivorous female insects prefer to oviposit on those host plants that are best for their offspring. Yet, past attempts to show the adaptiveness of host selection decisions by herbivores often failed. Here, we tested the PPH by including often neglected oviposition-induced plant responses, and how they may affect both egg survival and larval weight. We used seven Brassicaceae species of which most are common hosts of two cabbage white butterfly species, the solitary *Pieris rapae* and gregarious *P. brassicae*. Brassicaceous species can respond to *Pieris* eggs with leaf necrosis, which can lower egg survival. Moreover, plant-mediated responses to eggs can affect larval performance. We show a positive correlation between *P. brassicae* preference and performance only when including the egg phase: 7-day-old caterpillars gained higher weight on those plant species which had received most eggs*. Pieris* eggs frequently induced necrosis in the tested plant species. Survival of clustered *P. brassicae* eggs was unaffected by the necrosis in most tested species and no relationship between *P. brassicae* egg survival and oviposition preference was found. *Pieris rapae* preferred to oviposit on plant species most frequently expressing necrosis although egg survival was lower on those plants. In contrast to the lower egg survival on plants expressing necrosis, larval biomass on these plants was higher than on plants without a necrosis. We conclude that egg survival is not a crucial factor for oviposition choices but rather egg-mediated responses affecting larval performance explained the preference–performance relationship of the two butterfly species.

**Electronic supplementary material:**

The online version of this article (10.1007/s00442-019-04590-y) contains supplementary material, which is available to authorized users.

## Introduction

Host-plant selection for oviposition by insect females is a decisive step in establishing a new herbivore generation (Thompson 1988a, b; Thompson and Pellmyr 1991; Gripenberg et al. 2010). The preference–performance hypothesis (PPH) or ‘mother-knows-best’ hypothesis states that natural selection favors those insect females which prefer host plants where the offspring performs best, especially when immature stages are less mobile than adults. A good host plant is usually characterized either by a high nutritional value, reduced defense, and/or by enemy-free space (Mayhew 1997, 2001; Jaenike 1990; Craig and Ohgushi 2002; Gripenberg et al. 2010). In addition to host-plant quality and presence of natural enemies, various factors such as local host-plant abundance or distribution patterns of host plants shape oviposition preference and larval performance (Wiklund and Friberg 2008, 2009; Friberg et al. 2015). While the PPH is supported by many studies of butterflies and moths (Thompson 1988a, b; Thompson and Pellmyr 1991; Harris et al. 2001; Forister 2004; Forister et al. 2009), numerous others failed to do so (Thompson 1988a; Jaenike 1990; Mayhew 1997; Scheirs et al. 2000; König et al. 2016; Gripenberg et al. 2010).

Yet, the vast majority of innumerable laboratory studies testing the PPH did not consider that plants can activate defenses in response to egg deposition. Research has provided evidence that numerous plant species across highly diverse taxa defend against egg depositions of various insect species (Hilker and Fatouros 2015). Plants are capable of killing eggs (Fatouros et al. 2016). For example, egg-induced formation of a neoplasm (Petzold-Maxwell et al. 2011) or by hypersensitive response (HR)-like necrosis at the oviposition site (Shapiro and DeVay 1987; Fatouros et al. 2014; Griese et al. 2017) may result in egg detachment from the plant or egg dehydration. Furthermore, a plant can kill insect eggs by growing tissue that crushes the eggs (Karban 1983; Mazanec 1985; Aluja et al. 2004; Desurmont and Weston 2011). In addition, plants can receive insect eggs as early ‘warning cues’ of impending herbivory and reinforce or prime their defenses against the subsequently feeding larvae (Pashalidou et al. 2013, 2015c; Bandoly et al. 2015; Hilker and Fatouros 2015, 2016; Austel et al. 2016; Hilker et al. 2016). As a consequence, larval performance on initially egg-infested plants may be worse than on egg-free plants (Hilker and Fatouros 2016). Additionally, but less frequently shown so far, egg deposition can suppress plant defenses against larvae (Reymond 2013). The effects of egg deposition on subsequent plant defenses against larvae have been mainly overlooked until recently, with most studies on larval performance conducted by placing larvae onto an egg-free host plant (Hilker and Fatouros 2015, 2016).

The insect oviposition mode can have a significant impact on egg survival and larval performance. When eggs are laid in clusters, neonate larvae often show gregarious feeding behavior, which benefits offspring performance in some insect species (Denno and Benrey 1997; Clark and Faeth 1998; Fordyce 2003; Allen 2010; Desurmont et al. 2014; Martínez et al. 2017). On the other hand, many herbivorous insects lay single eggs, spreading them over a larger area, possibly as a means of reducing predation risk and competition (Root and Kareiva 1984; Nufio and Papaj 2001). Egg-induced plant defense affecting larval performance is especially known for insect species laying eggs in clutches (Hilker and Fatouros 2015). However, plant response to singly laid eggs of *Manduca sexta* reinforces the defense against *M. sexta* larvae (Bandoly et al. 2016). It remains to be elucidated whether the egg-laying mode (single eggs vs. egg clutches) affects egg-induced plant defense targeting the eggs and how this in turn depends on the plant species receiving the eggs.

The aim of this study is to elucidate whether there is a positive outcome of the PPH when not only considering relationships between oviposition preference and larval performance, but also when including egg survival rates and egg-induced changes in plant suitability for feeding caterpillars. Therefore, we investigated oviposition preference, egg survival and larval performance of *Pieris brassicae* and *P. rapae* on seven Brassicaceae species. Pierid butterflies have co-evolved over 90 million years with their host plants in the order Brassicales (Wheat et al. 2007; Edger et al. 2015). Both butterfly species are known to use various wild and cultivated brassicaceous plants as hosts (Feltwell 1982; Chew and Renwick 1995; Gols et al. 2011), whereby *P. rapae* can include also non-brassicaceous plants in their diet (Friberg et al. 2015). *Pieris* caterpillars are well adapted to Brassicaceae by their ability to detoxify glucosinolates, plant secondary metabolites characteristic for this plant taxon (Hopkins et al. 2009). While *P. rapae* lays single eggs on plants, *P. brassicae* lays egg clutches containing up to 200 eggs (Feltwell 1982) (Supplementary Figure S1). Egg deposition by these Pierid species is known to induce an HR-like leaf necrosis in several host plant species (Fatouros et al. 2016). Additionally, previous egg deposition by *P. brassicae* on brassicaceous plant species negatively affects larval performance (Geiselhardt et al. 2013; Pashalidou et al. 2013, 2015a; Bonnet et al. 2017). It remains unknown so far how egg deposition of the conspecific solitary species *P. rapae* affects subsequently feeding larvae through egg-mediated plant responses.

We specifically addressed the following questions: (1) is oviposition choice of two pierid species, *P.brassicae* and *P. rapae* affected by the plant species’ capability to activate an egg-killing response (i.e., HR-like necrosis)? (2) Do females prefer to oviposit on plants on which their eggs show highest survival rates and larvae perform best? (3) Do plant-mediated effects of eggs change caterpillar performance (i.e., defense priming) and if so (4) do they correlate with butterfly oviposition choices?

## Material and methods

### Insects and plants

*Pieris brassicae* L. and *P. rapae* L. (Lepidoptera: Pieridae) were reared since many generations in a climatized room (21 ± 1 °C, 50–70% RH, L16:D8) on *B. oleracea* var. *gemmifera* L. plants. Female butterflies mated 2–3 days after eclosion. Their oviposition preference was tested 2 days after mating. The females have a high egg load at this age and mating status (David and Gardiner 1962).

Eight different brassicaceous species were used in a preference experiment, seven in a performance experiment. Apart from *Raphanus sativus* L., all plant species were non-domesticated species. We obtained *R. sativus* from De Bolster seed company (The Netherlands), *Hirschfeldia incana* L. Lagr.-Foss. from the US, California, *Brassica nigra* L. from the Centre of Genetic Resources (CGN, Wageningen, the Netherlands) from an early flowering accession (CGN06619), *Sinapis arvensis* L. from Vlieland, in the north of The Netherlands, *B. montana* Pourr. from CGN (CGN18472 accession from Italy), *B. rapa* L. from Binnenveld, west of Wageningen (The Netherlands) and *B. oleracea* L. ‘Kimmeridge’ from the south coast of England. Most of the tested plant species are frequently used as host plants by both species in the Netherlands, especially *B. nigra, B. rapa, B. oleracea* and *S. arvensis*, except for *A. thaliana* (Fei et al. 2014; Harvey et al. 2007). Natural accessions of *A. thaliana* are visited by pierids in the North of Europe (Wiklund 1984). *Hirschfeldia incana* was shown to be a host plant for *P. rapae* in California (Shapiro 2002). *Brassica montana* is a wild ancestor of *B. oleracea*; both species grow in coastal regions either in Southern Europe or Britain, respectively. *Arabidopsis thaliana* (Col-0) was used only for the preference tests. Because of its small size, it was excluded from performance studies, as more than just the focal egg-induced plant would be needed to feed the caterpillars. All plants were in the non-flowering stage when tested, except *A. thaliana*, which already flowered. All plants were cultivated in pots filled with standard potting soil and watered daily; no fertilization was added. They were grown in a climate room (18 ± 4 °C, 60–80% RH, L16:D8). To use plants of similar biomass in the bioassays, *B. oleracea* was 4 weeks old, and all other plants were 3 weeks old at the time of infestation.

### Butterfly oviposition preference

To determine which plant species is preferred for oviposition, we simultaneously offered the above-mentioned eight plant species to a mated female butterfly. An individual plant of each species was placed into a mesh cage (75 × 75 × 115 cm). The plants were set up in a circle with the leaves not touching each other. The design was a randomized block with 18 replicates per butterfly species. The two butterfly species were tested in separate cages at different time points in a greenhouse compartment (23 ± 5 °C, 50–70% RH, L16:D8). After placing the plants inside a cage, one mated female butterfly was released. The number of *P. rapae* eggs or *P. brassicae* egg clutches, respectively, was counted on each plant 3 h after release of the butterfly. Preliminary experiments showed that most butterfly females will make an oviposition choice within this time period.

### Plant treatments for performance tests

To assess egg survival rates and performance of larvae on previously egg-deposited plants, a plant individual of each species was infested with either *P. rapae* or *P. brassicae* eggs. Each plant was placed in a cage with approximately 100 butterflies, located in a climate room (21 ± 1 °C, 50–70% RH, L16:D8). The first fully developed leaf of each plant (fourth or fifth from the top) was exposed to either *P. brassicae* or *P. rapae* butterflies for egg deposition, while the rest of the plant was covered with a fine mesh. We limited the number of eggs deposited onto a plant to 20 eggs of *P. brassicae* (laid in a clutch) and to eight single *P. rapae* eggs per plant. Limiting egg deposition was done by observing the butterflies after introduction into the cages and removing them as soon as they had deposited the mentioned number of eggs. Those numbers were chosen to mimic naturally occurring egg numbers per plant (Feltwell 1982; Fatouros et al. 2014). Occasionally, extra laid eggs were immediately removed using a fine brush [see Pashalidou et al. (2013) for details]. In total, seven to nine individual plants per species were infested with *P. brassicae* eggs, and six to seven individual plants per species received *P. rapae* eggs.

### Plant response to egg deposition, egg mortality and larval performance

To determine egg survival, we counted the number of larvae hatching from the twenty (*P. brassicae*) or eight (*P. rapae*) eggs deposited on a plant. After 4 days, presence/absence of HR-like necrosis was scored as previously described by Griese et al. (2017). After 5 days, survival of eggs was noted by counting the number of hatched caterpillars. To assess larval performance and the impact of the plant’s response to previous egg deposition on larval performance, we divided the neonate larvae hatching from egg-deposited plants into two groups. Half of them were placed back onto the previously egg-infested plant (labeled ‘egg and feeding’, EF) (on the adaxial side of the leaf where they hatched), and the other half was transferred to an egg-free plant (labeled ‘feeding’, F) plant of the same species and placed onto the adaxial side of the leaf as well. Three and seven days after hatching, caterpillar weight was measured on a microbalance (accuracy = 1 µg; Sartorius AG, Göttingen, Germany). We weighed each caterpillar individually, and afterwards the caterpillars were transferred back to their original position, on EF or F plants. Every EF and F plant was considered one replicate.

### Statistical analysis

Data on *P. rapae* oviposition preference for host plants were analyzed by a generalized linear model (GLM) (Poisson distribution), with plant species as fixed factor and the number of eggs per plant as response variable. The post hoc analysis was performed using a linear hypothesis test (multcomp package). Because *P. brassicae* laid most eggs in a single clutch each time, only oviposition ‘yes’ or ‘no’ was scored when determining oviposition preference. These data were analyzed by a GLM (binomial distribution) with the plant species as fixed factor, and the presence/absence of oviposition as response variable (post hoc test: linear hypothesis test).

Data on egg survival of each butterfly species were analyzed by a generalized linear mixed effect model (GLMM, lme4 package) with binomial distribution. The model included egg survival as response variable, and plant species, presence/absence of HR as well as the interaction between both variables were fixed factors. Date of infestation were a random factor. A post hoc analysis was conducted using linear hypothesis tests for plant species and interaction terms.

To evaluate whether oviposition preference for a plant species can be linked to egg survival, we ran a correlation analysis using Spearman correlation as well as linear regression to generate regression lines. We conducted this analysis first by relating the proportion of eggs (or egg clutches) laid on each plant species with the proportion of eggs surviving on each plant species. To elucidate the relationship between the plant’s ability to express HR-like necrosis and egg survival, we correlated the proportion of eggs laid on each plant species to the proportion of eggs surviving on those plants, which expressed HR-like necrosis in response to the eggs. To elucidate the relationship between the plant´s ability to express HR-like necrosis and oviposition preference, we linked the proportion of eggs or egg clusters laid to the proportion of plants expressing HR.

Data on caterpillar weight obtained on all plant species (subjected to prior egg deposition or not) were analyzed using linear mixed effect models (LMM). We calculated the average caterpillar weight per plant. The logarithm of the mean caterpillar weight 3 or 7 days after hatching was the dependent variable, plant species and egg infestation as well as the interaction between them were used as independent variables, the random factor was the date of egg infestation. A post hoc analysis was conducted using linear hypothesis tests for plant species and interaction terms. The effect of HR-like necrosis on caterpillar weight was tested using the subset of plants infested with eggs and performing LMM on the logarithmic data of the mean caterpillar weight. Expression of HR-like necrosis, plant species as well as the interaction between both factors was included in the model. A post hoc analysis was conducted using linear hypothesis tests for plant species and interaction terms.

To detect possible links between butterfly oviposition preference and performance of 3- or 7-day-old caterpillars, a linear regression analysis was conducted. We conducted this analysis first by relating the proportion of eggs laid on each plant species with the weight of caterpillars on each plant species. Furthermore, we ran an analysis by relating the proportion of eggs laid on each plant species to the weight of caterpillars on (1) plants which received eggs prior to larval feeding (EF) and (2) plants which were left without any eggs (F); thus, we aimed to test the hypothesis that females prefer to oviposit on plants with most modest (for *P. rapae:* putative) egg-mediated reinforcement of defense against the larvae. In addition, we analyzed the relationship between caterpillar weight and the proportion of eggs laid on plants expressing HR-like necrosis; thus, we aimed to test the hypothesis that females prefer to oviposit on plants whose HR-like necrosis has the most modest effect on the performance of their offspring. Finally, we tested the relationship between the proportion of plants expressing HR-like necrosis and caterpillar weight. Thus, we aimed to gain insight into whether the frequency of HR-like necrosis in response to the eggs relates to caterpillar weight.

All analyses were performed using R 3.3.2 (R Core Team 2016).

## Results

### Oviposition preference

#### Gregarious species

**Fig. 1:**
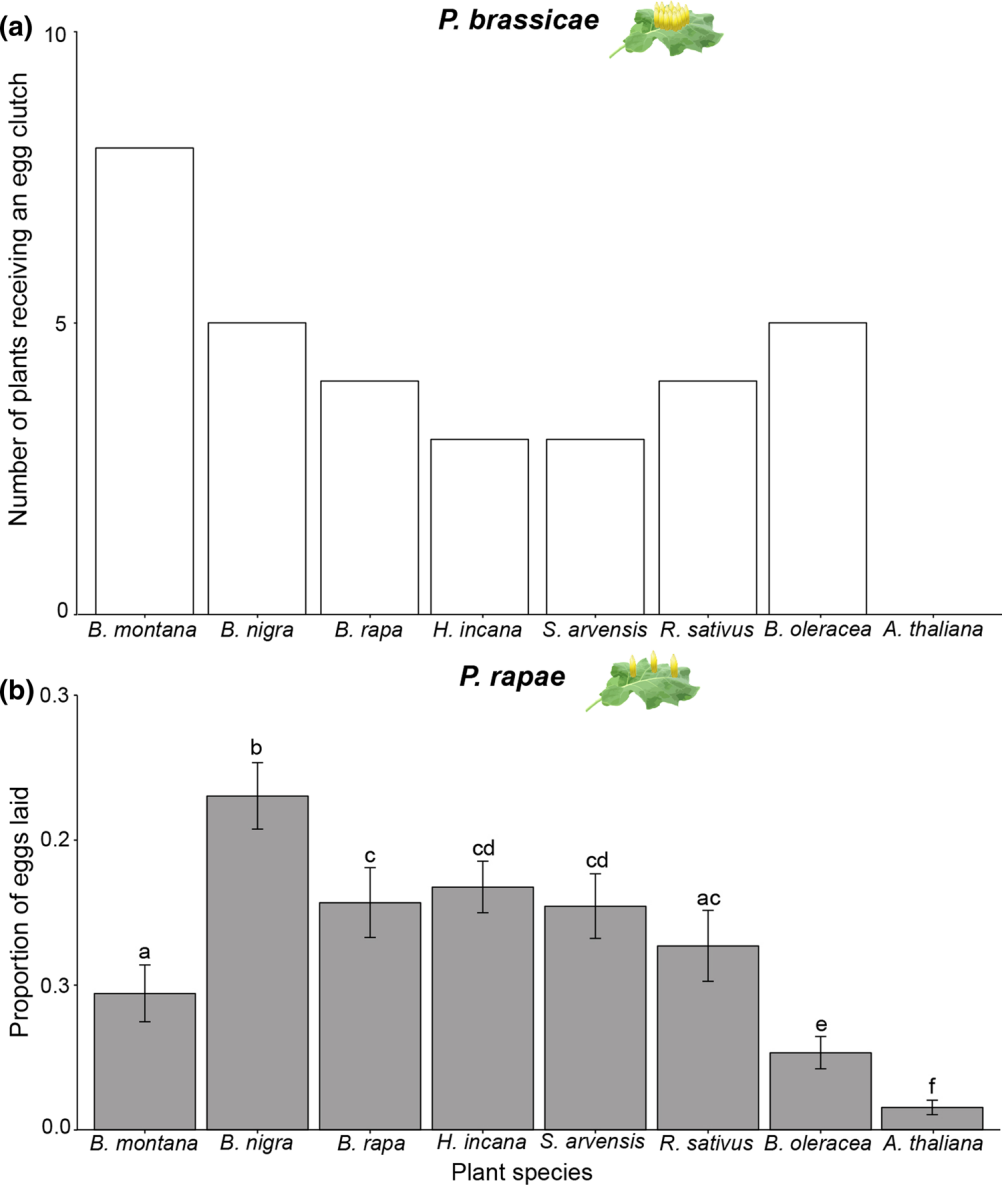
Oviposition preference bioassay. **a** Number of plants, which received an egg deposition by *Pieris brassicae.* Female *P. brassicae* always laid a maximum of one egg clutch per plant; therefore, number of egg-deposited plants is presented. **b** Proportion of eggs laid by *P. rapae* on different brassicaceous plant species. The number of single eggs of *P. rapae* was counted per plant species. Mean proportion ± SE is given. In total, 18 plants per species were tested in random setups. Small letters indicate significant differences between plant species with *P* < 0.05, GLM

Although *P. brassicae* females distributed their egg clutches unevenly between plants (*χ*^2^ = 19.65, df = 7, *P* = 0.04, GLM, Fig. 1a), a post hoc test did not reveal a significant preference (Supplementary Table 1). *Arabidopsis thaliana,* which was in the flowering stage (in contrast to all other plant species), did not receive any egg clutches from *P. brassicae* in this setup*.* Oviposition choices of *P. brassicae* did not correlate with plant fresh weight (*S* = 1720.7, *ρ* = 0.25, *P* = 0.24, Spearman correlation, Supplementary Figure S2).

#### Solitary species

*Pieris rapae* females significantly preferred to oviposit on *B. nigra* over all other seven simultaneously offered plant species (Supplementary Table 1). The plant species chosen least frequently for oviposition were *B. oleracea* and *A. thaliana* (*χ*^2^ = 292.67, *df* = 7, *P* ≤ 0.001, GLM, Fig. 1b). The oviposition preference of *P. rapae* did not correlate with plant fresh weight (*S* = 1897.2, *ρ* = 0.18, *P* = 0.41, Spearman correlation, Supplementary Figure S2).

### Egg survival and effect of plant species and HR-like necrosis

#### Gregarious species

**Fig. 2:**
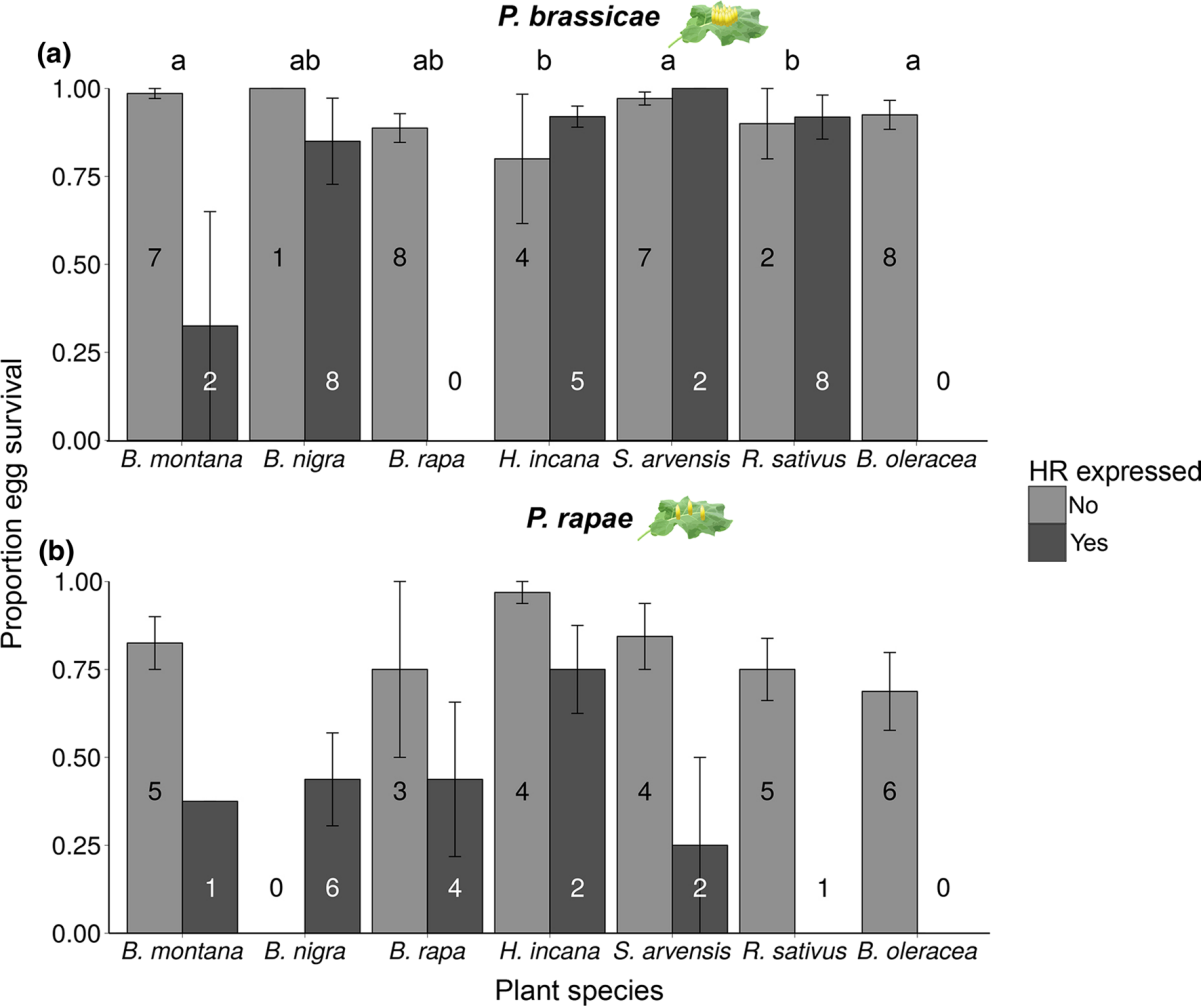
Effect of HR-like necrosis on survival of eggs of two *Pieris* species on different brassicaceous plant species (mean proportion ± SE). Numbers given in the bars indicate the number of plants. Different letters indicate significant differences (*P* < 0.05, GLM) between plant species regardless of HR-like necrosis. **a** Proportion survival of *P. brassicae* eggs, with each egg clutch consisting of 20 eggs. **b** Proportion survival of *P. rapae* eggs, with eight eggs being laid per plant

Plant species significantly affected *P. brassicae* egg survival (*χ*^2^ = 20.39, *df *= 6, *P* = 0.002, GLMM, Fig. 2a). Egg survival was highest on *B. montana* chosen most for oviposition, while it was lowest on *H. incana* and *R. sativus* compared to all other plants (apart from *B. nigra*) (Supplementary Table S2). Five out of the seven tested plant species expressed an HR-like necrosis in response to *P. brassicae* eggs (Fig. 2a, Supplementary Table S2). Overall, induction of HR-like necrosis did not significantly affect egg survival (*χ*^2^ = 0.41,* df* = 1, *P* = 0.52, GLMM), while the effect of HR-like necrosis on survival of *P. brassicae* eggs was plant species specific (*χ*^2^ = 30.83,* df* = 4, *P* < 0.001, GLMM). On *B. montana,* egg survival was much lower on the two HR-expressing plants than on the seven non-HR plants (Fig. 2a, Supplementary Table S3–4).

#### Solitary species

The plant species selected by *P. rapae* females did not significantly affect egg survival (*χ*^2^ = 11.19,* df* = 6, *P* = 0.08, GLMM, Fig. 2b). Six out of the seven tested plant species expressed HR-like necrosis induced by *P. rapae* eggs (Fig. 2b, Supplementary Table S2). A significantly higher proportion of *P. rapae* eggs survived on non-HR plants compared to plants expressing HR-like (*χ*^2^ = 13.58,* df* = 1, *P* < 0.001, GLMM). This effect of *P. rapae* egg-induced HR-like necrosis on egg survival was—in contrast to the *P. brassicae* egg-induced response—independent of the plant species (*χ*^2^ = 4.43,* df* = 4, *P* = 0.35, GLMM) (Fig. 2b, Supplementary Table S3–4).

### Correlation between oviposition preference and egg survival

**Fig. 3:**
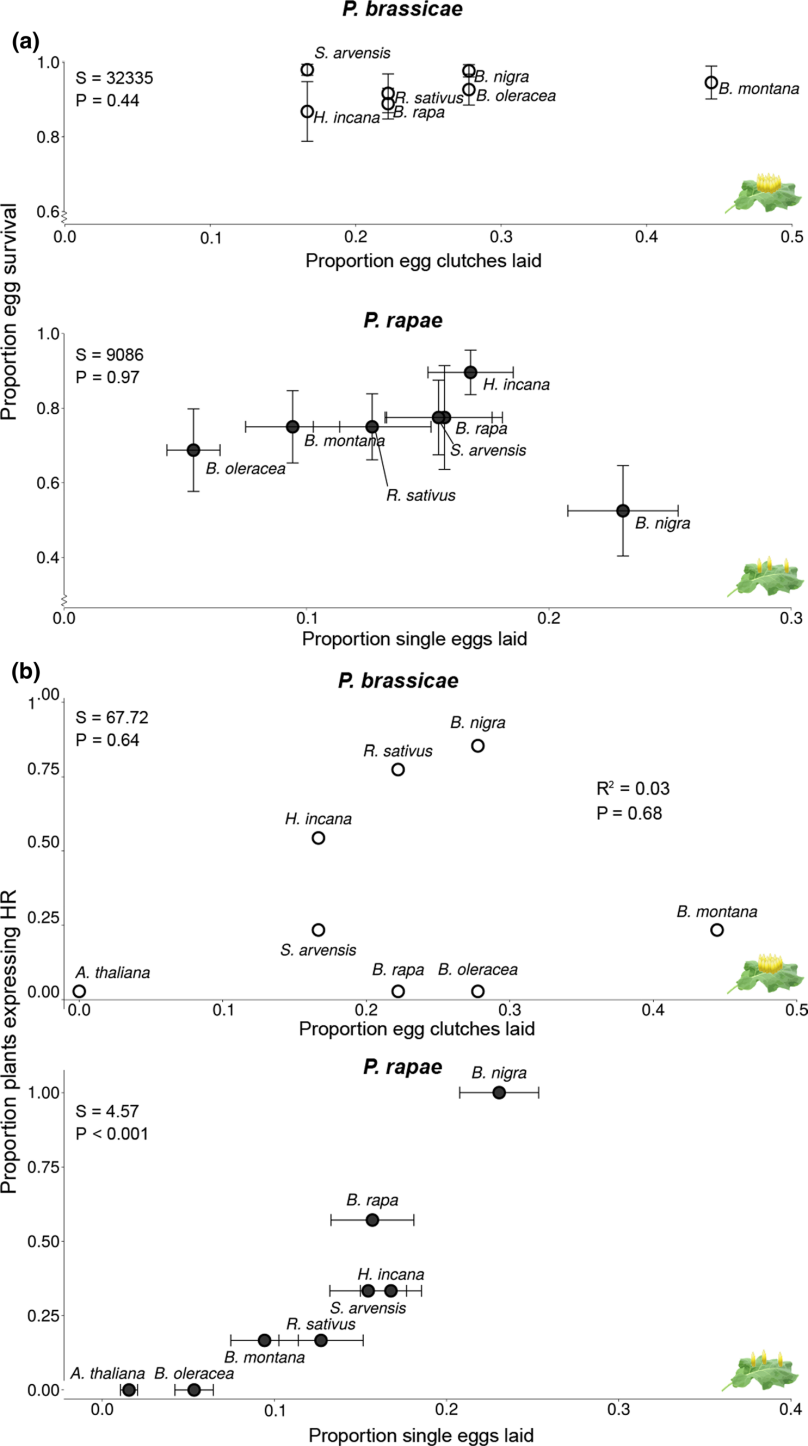
Correlation between proportion of eggs laid on different brassicaceous plants by two *Pieris* butterflies and **a** proportion of egg survival (mean ± SE) or **b** proportion of HR-like necrosis (mean ± SE). Text boxes show correlation results. Proportion of HR + plants has no error bars, and for *P. brassicae,* no error bars for preference are available. *N* = 8—10 plants for *P. brassicae*, *N* = 5—7 plants for *P. rapae*

We did not detect a significant correlation between oviposition preference and survival of the eggs for either of the two butterfly species (proportion of eggs laid related to egg survival; Spearman correlation; for *P. brassicae*: *S* = 32,335, *ρ* = 0.10, *P* = 0.44, for *P. rapae*: *S* = 9086.3, *ρ* = 0.01, *P* = 0.97, Fig. 3a). However, the proportion of *P. rapae* eggs laid was positively correlated with the proportion of plants expressing HR-like necrosis against those eggs (*S* = 4.57, *ρ* = 0.96, *P* < 0.001, Spearman correlation, Fig. 3b)*.* For *P. brassicae,* this correlation was not significant (*S* = 67.72, *ρ* = 0.19, *P* = 0.64, Spearman correlation, see Fig. 3b).

### Effect of plant species, egg infestation and HR on larval performance

#### Gregarious species

**Fig. 4:**
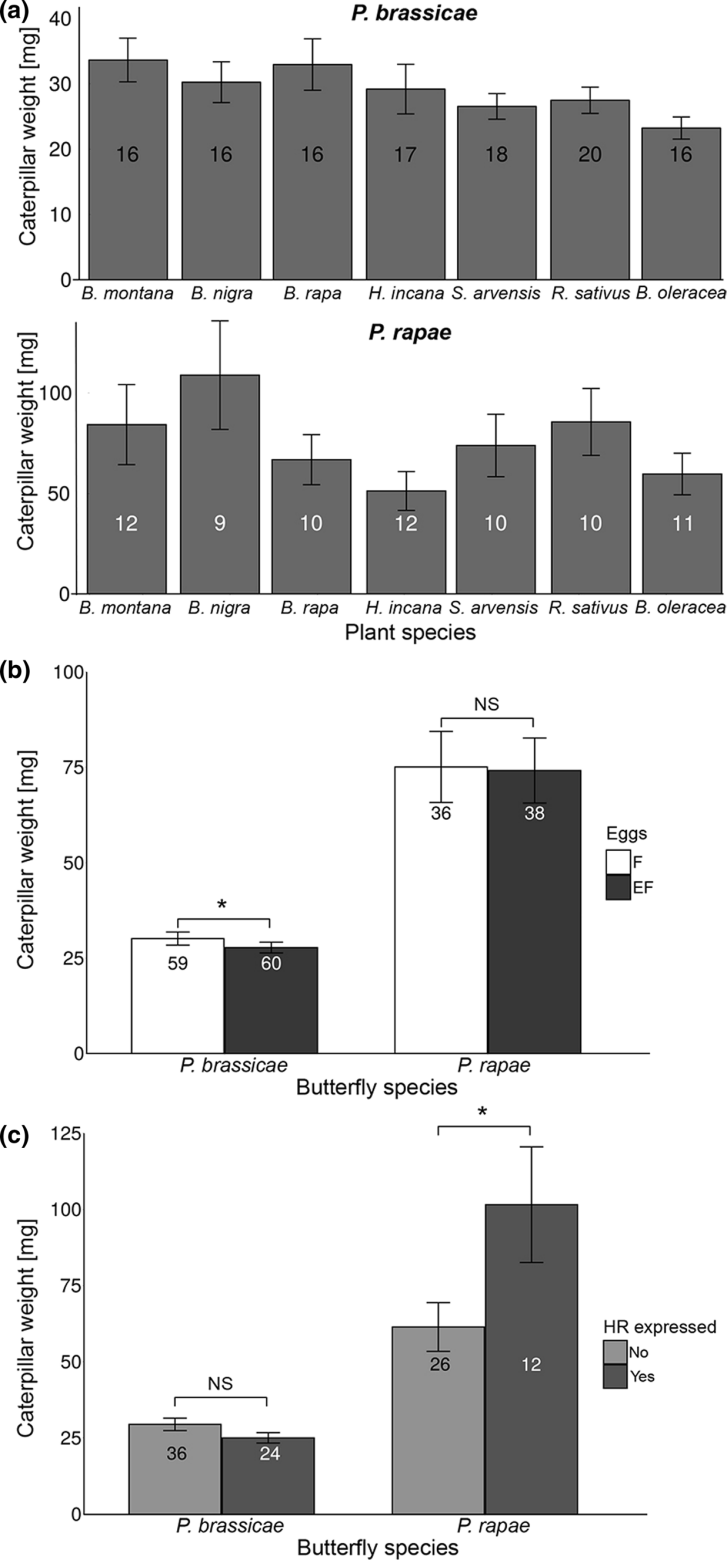
Effects of plant species (**a**), egg-mediated defenses (**b**) or leaf necrosis (**c**) on weight (mean ± SE) of 7-day-old *Pieris brassicae* or *P. rapae* caterpillars. For **a** weights of caterpillars feeding upon egg-free and previously egg-deposited plants are pooled, **b** weights of caterpillars feeding upon egg-free (F) and previously egg-deposited plants (EF) are shown separately, and **c** weights of caterpillars feeding upon previously egg-deposited plants are shown separately by the presence/absence of egg-induced HR-like necrosis. Numbers in the bars represent the number of plants within the group. The weight of caterpillars was averaged per plant. Asterisks indicate significant differences. *P < 0.05, *ns* not significant, GLMM

The weight of 7-day-old *P. brassicae* caterpillars did not vary significantly depending on the plant species they were feeding on (*χ*^2^ = 12.44,* df* = 6, *P* = 0.05, LMM, Fig. 4a). However, the plants’ response to prior egg deposition significantly affected performance of *P. brassicae* larvae. Seven-day-old larvae developing on plants that previously had received eggs (EF) significantly gained less weight than those on plants that had not received eggs (F) (*χ*^2^ = 5.27,* df* = 1, *P* = 0.02, LMM, Fig. 4b). Caterpillars feeding on plants without prior egg deposition gained about 5% more weight. This egg-mediated effect on performance was independent of the plant species (no interactive effect between plant species and egg infestation on larval weight; *χ*^2^ = 2.51,* df* = 6, *P* = 0.87, LMM). HR-like necrosis induced by previously laid eggs did not affect the weight of caterpillars (*χ*^2^ = 0.72,* df* = 1, *P* = 0.40, LMM, see Fig. 4c), and neither did plant species nor did the interaction between plant species and HR-like necrosis (*χ*^2^ = 5.76,* df* = 6, *P* = 0.45 and *χ*^2^ = 5.46,* df* = 4, *P* = 0.24, LMM). The weight of 3-day-old caterpillars is presented in the supplementary material (Supplementary Figure S3).

#### Solitary species

When considering 7-day-old *P. rapae* caterpillars on both egg-free and previously egg-deposited plants, their weight was not affected by the plant species they were feeding on (*χ*^2^ = 5.04,* df* = 6, *P* = 0.54; LMM, Fig. 4a). When excluding the occurrence of HR-like necrosis induced by egg deposition, egg infestation preceding larval feeding did not affect larval weight (*χ*^2^ = 0.001,* df* = 1, *P* = 0.97; LMM, Fig. 4b). Neither did the interaction between egg infestation and plant species affect larval weight (*χ*^2^ = 1.09,* df* = 6, *P* = 0.98, LMM, Fig. 4c). Yet, larvae feeding on EF plants expressing an HR-like necrosis were significantly heavier than those feeding on EF plants that did not show HR-like necrosis (*χ*^2^ = 4.14,* df* = 1, *P* = 0.04, LMM, Fig. 4c). Neither plant species nor the interaction between plant species and HR-like necrosis affected caterpillar weight on previously egg-infested plants (*χ*^2^ = 3.73,* df* = 6, *P* = 0.71 and *χ*^2^ = 3.93,* df* = 3, *P* = 0.27, LMM). The weight of 3-day-old caterpillars is presented in the supplementary material (Supplementary Figure S3).

### Correlation between oviposition preference and larval performance

To assess whether there was a correlation between adult oviposition preference and larval performance, we first analyzed the relationship between the proportion of eggs laid and the weight of three (Supplementary information and Supplementary Figure S4) or 7-day-old caterpillars feeding on previously oviposited EF plants and egg-free F plants for each plant species.

#### Gregarious species

**Fig. 5:**
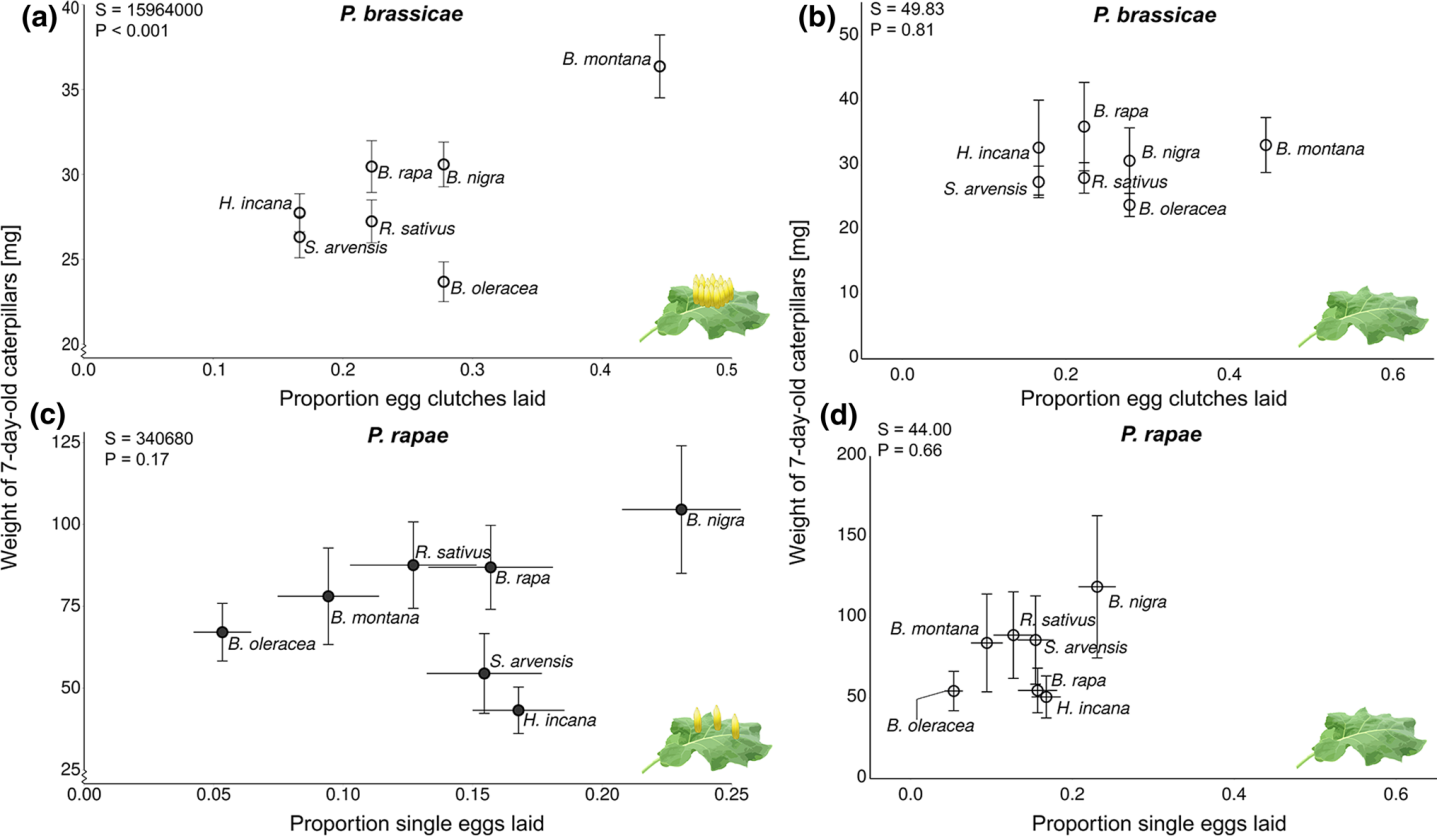
Correlation between oviposition preference and larval performance of 7-day-old *Pieris* caterpillars on different previously egg-deposited (EF) (**a**, **b**) or egg-free (F) (**c**, **d**) brassicaceous plant species. Caterpillar weight (mean ± SE) and proportion of eggs/egg clusters laid are shown. Results of the Spearman correlation test are shown in text boxes. *Y*-axes do not start at zero to show graph in larger detail

Weight of 7-day-old *P. brassicae* larvae significantly and positively correlated with the number of eggs laid. Seven-day-old *P. brassicae* larvae were the heaviest on those plant species that received most egg clusters (*S* = 15,964,000, *ρ* = 0.17, *P* < 0.001, Spearman correlation, Fig. 5a). The proportion of egg clusters laid did not correlate with the weight of caterpillars feeding on egg-free plants (*S* = 34, *ρ* = 0.39, *P* = 0.40, Spearman correlation, Fig. 5b).

When considering the weight of 7-day-old *P. brassicae* caterpillars with respect to the plant’s capability to express HR in response to the eggs, weight of caterpillars feeding on previously egg-deposited HR-expressing  plants did not correlate with the proportion of egg clutches per plant (*S* = 22, *ρ* = − 0.1, *P* = 0.95, Spearman correlation). Neither was a correlation found between the proportion of plants expressing HR-like necrosis in response to oviposition and the caterpillar weight (*S* = 36.65, *ρ* = 0.35, *P* = 0.45, Spearman correlation).

#### Solitary species

In contrast to *P. brassicae,* the weight of 7-day-old *P. rapae* larvae feeding on previously egg-infested plants neither correlated with the proportion of eggs laid (*S* = 340,680, *ρ* = 0.13, *P* = 0.17, Spearman correlation, Fig. 5c) nor with egg load when larvae were feeding on egg-free plants (*S* = 50, *ρ* = 0.11, *P* = 0.84, Spearman correlation, Fig. 5d). Weight of 7-day-old caterpillars feeding on HR + plants did not correlate with the proportion of eggs laid (*S* = 32, *ρ* = − 0.6, *P* = 0.35, Spearman correlation). Furthermore, the weight of 7-day-old larvae was not correlated with the proportion of plants expressing HR-like necrosis (*S* = 29.52, *ρ* = 0.47, *P* = 0.28, Spearman correlation).

## Discussion

We show that plant-mediated effects of egg deposition partly explain the positive relationship between the proportion of eggs laid and larval weight. Seven-day-old caterpillars of *P. brassicae* gained most biomass on the plant species that received most eggs (*B. montana*) only when including the effect of previous egg deposition. However, oviposition choices of both *Pieris* species did not correlate with egg survival. Singly laid *P. rapae* eggs always induced HR-like leaf necrosis in *B. nigra* on which egg survival was the lowest. Nevertheless, the solitary butterfly deposited most eggs on this plant species. Larval biomass of *P. rapae* was higher on plants expressing egg-induced HR-like necrosis compared to plants without necrosis. In contrast, the gregarious *P. brassicae* showed no significant oviposition preference for any of the tested plant species, and egg survival was hardly affected by HR-like necrosis. However, weight of *P. brassicae* caterpillars feeding on previously egg-deposited plants was lower than of those feeding on egg-free plants.

Females of the solitary species *P. rapae* laid most eggs on *B. nigra* plants on which egg survival was lowest but this might not be considered an “oviposition mistake” (Larsson and Ekbom 1995). Oviposition choices are influenced by different cues over long and short distances (Schoonhoven et al. 2005). Cues that signal intraspecific variation in suitability might be absent or of low detectability (Larsson and Ekbom 1995). Most plants show phenotypic variation in the expression of HR-like necrosis and it might be difficult for the butterflies to discriminate between egg-resistant (HR+) and egg-susceptible (HR−) genotypes. Yet, a better larval performance might trade-off with a low egg survival. Weight of *P. rapae* caterpillars was highest on *B. nigra* plants expressing HR where egg survival was reduced most. High larval hatching rates on plants with high egg load might result in several problems for the larvae, i.e., fast food depletion, easy detectability of caterpillars by parasitoids, increased cannibalism and the spread of pathogens (Prokopy and Roitberg 2001).

Similar preference and performance correlations were obtained in studies with the polyphagous *Anastrepha ludens* fruit fly in response to six different host plants belonging to different families. The second most preferred plant species for oviposition, *Casimiroa edulis* (white sapote), was also the host on which larvae performed best. However, approximately half of all egg clutches laid on *C. edulis* were killed by a wound tissue growth response that led to egg encapsulation (Aluja et al. 2004; Birke and Aluja 2018). We suggest that *P. rapae* butterflies can afford laying most eggs on the most nutritive plants, because a nutritionally high-quality plant can harbor numerous larvae of the solitary species without competing for resources. While survival rate of *P. rapae* eggs on the most preferred host *B. nigra* was lowest, survival rate of *P. brassicae* eggs on the plant species on which most eggs were laid (*B. montana*) was similar to the plant species that received fewer eggs. This suggests that *P. brassicae* does not adjust its oviposition choices to egg survival rates. *Pieris brassicae* can afford to be less choosy when selecting a host plant for oviposition because gregariously laid eggs have certain advantages with regard to egg survival over singly laid eggs. Gregariousness may, for example, contribute to protection from desiccation inflicted by the necrosis (Stamp 1980; Clark and Faeth 1998; Griese et al. 2017).

A positive relationship was found between plants expressing HR-like leaf necrosis induced by singly laid *P. rapae* and the oviposition preference of *P. rapae.* No such relationship was found between oviposition choices of *P. brassicae* and the occurrence of HR-like leaf necrosis in the various tested plant species. It is possible that those *P. rapae* larvae hatching from eggs despite HR-like necrosis also face less competition, eventually leading to heavier larvae than on non-HR plants. Another possibility could be that caterpillars perform best on those plant species showing strong HR-like necrosis, because these plants provide high nutritional quality. Based on a meta-analysis, Wetzel et al. (2016) suggested that host plant nutritional quality might be more important for offspring performance than plant defenses against larvae. For example, relationships between plant nutrients and performance of noctuid caterpillars (*Heliothis virescens* and *Helicoverpa zea*) were consistently concave down, while the relationships between plant defenses and herbivore performance were close to linear.

Plant-mediated effects of eggs on caterpillar performance were only shown in the case of the gregarious *P. brassicae,* but not for the solitary *P. rapae.* When comparing weight of caterpillars on egg-free and previously egg-deposited plants (all species), *P. brassicae* caterpillars gained less weight on the latter. These results confirm previous results on the performance of *P. brassicae* tested on *A. thaliana* (Geiselhardt et al. 2013), *B. nigra* (Bonnet et al., 2017; Pashalidou et al. 2013, 2015a, b) or *S. arvensis* and *B. oleracea* (Pashalidou et al. 2015a). Similarly, reinforced plant defense against insect larvae mediated by prior egg deposition occurs in several interactions between plants and insects that lay eggs in clutches and feed gregariously during larval development, namely *Diprion pini* sawflies on pine (Beyaert et al. 2012); *Spodoptera littoralis* caterpillars on wild tobacco*,* (Bandoly et al. 2015, 2016) and *Xanthogaleruca luteola* leaf beetles on elm (Austel et al. 2016). Singly laid eggs of *P. rapae* did not prime plant defense against caterpillars. The reason why only plant responses to egg clutches of the gregarious *P. brassicae* negatively affected the performance of subsequently feeding caterpillars and not the singly laid eggs of *P. rapae* remains to be investigated.

The differences in preference and performance recorded for the two butterfly species might be due to their different oviposition and feeding modes. Unlike for singly laid eggs (*P. rapae*), survival of clustered egg (*P. brassicae*) was not lower on plants expressing HR-like necrosis. This confirms a previous study where survival of *P. brassicae* eggs on *B. nigra* expressing HR-like necrosis was not affected even under field conditions (Griese et al. 2017). However, when *P. brassicae* eggs were experimentally kept singly, mortality increased. Single eggs suffered more from drops in humidity than a cluster of five eggs (Griese et al. 2017). Humidity drops are likely a characteristic of necrotic leaf tissue and a possible reason for egg-killing (Shapiro and DeVay 1987). Hence, our current study supports the assumption that egg-induced HR-like necrosis negatively affects survival of singly laid eggs (as those of *P. rapae*) rather than clustered eggs (as those of *P. brassicae).* This is further supported by previous studies which showed that single eggs of *P. rapae* as well as of *P. napi* suffer high mortality when the host plant expresses HR-like necrosis (Shapiro and DeVay 1987; Fatouros et al. 2014).

With respect to our initial questions, our study has shown that *P. brassicae* laid most eggs on those plant species which provide the best larval performance, while these plant species do not provide best egg survival rates. The plant species’ capability to activate HR-like necrosis in response to egg deposition affected the oviposition choice of *P. rapae* in so far, as this butterfly species laid most eggs on a plant species where HR expression frequently occurred, and egg survival rates were low. This behavior might be considered a counter-adaptation because a high egg load on a plant with a high egg-killing capability ensures survival of at least some offspring. Since survival of eggs of *P. brassicae* was not affected by the plant’s HR-like, this butterfly species can afford to lay many eggs on a plant species with high HR-expression frequency. Our data indicate that the gregarious oviposition mode of *P. brassicae* allows this butterfly species to be less choosy than *P. rapae* in selecting an oviposition site because the gregariously laid eggs are not affected by HR.

Future studies need to further address the question whether the differences in the effects of plant responses to these two Pierid species are due to the different oviposition modes and larval feeding behaviors (singly vs. gregariously) or whether other insect species-specific traits are important as well. Moreover, our results on positive oviposition preference–larval performance relationships and effects of the egg phase therein were mainly driven by two plant species. Therefore, we suggest to further test the PPH with a different or more numerous set of plant species to gain a better understanding of how general these effects are. Previous studies have shown that egg-mediated priming affects several performance traits apart from larval weight, i.e., developmental time, pupal weight and larval survival (Pashalidou et al. 2015a; Hilker and Fatouros 2015, 2016). Yet, it remains to be investigated whether oviposition preference correlates with these other performance measures. Furthermore, future studies need to explore whether clustering of eggs by *P. brassicae* and a high number of singly laid eggs for *P. rapae* may evolved as counter adaptations against the egg-killing leaf necrosis. Other countermeasures of pierid butterflies against egg-induced plant defenses include oviposition on inflorescence stems instead of leaves, as occurs in some other pierid species feeding on Brassicaceae (*P. napi* and *Anthocharis cardamines*) (Griese et al. 2019) and feeding preference for flowers when they are available (*A. cardamines* and *P. brassicae*) (Wiklund and Åhrberg 1978; Smallegange et al. 2007).

## Electronic supplementary material

Below is the link to the electronic supplementary material.

Supplementary file1 (DOCX 1289 kb)
